# Identification of the putative binding pocket of valerenic acid on GABA_A_ receptors using docking studies and site‐directed mutagenesis

**DOI:** 10.1111/bph.13329

**Published:** 2015-10-25

**Authors:** D Luger, G Poli, M Wieder, M Stadler, S Ke, M Ernst, A Hohaus, T Linder, T Seidel, T Langer, S Khom, S Hering

**Affiliations:** ^1^ Department of Pharmacology and ToxicologyUniversity of ViennaViennaAustria; ^2^ Department of PharmacyUniversity of PisaPisaItaly; ^3^ Department of Pharmaceutical ChemistryUniversity of ViennaViennaAustria; ^4^ Department of Molecular Neurosciences, Center of Brain ResearchMedical University of ViennaViennaAustria

## Abstract

**Background and Purpose:**

β2/3‐subunit‐selective modulation of GABA_A_ receptors by valerenic acid (VA) is determined by the presence of transmembrane residue β2/3N265. Currently, it is not known whether β2/3N265 is part of VA's binding pocket or is involved in the transduction pathway of VA's action. The aim of this study was to clarify the localization of VA's binding pocket on GABA_A_ receptors.

**Experimental Approach:**

Docking and a structure‐based three‐dimensional pharmacophore were employed to identify candidate amino acid residues that are likely to interact with VA. Selected amino acid residues were mutated, and VA‐induced modulation of the resulting GABA_A_ receptors expressed in *Xenopus* oocytes was analysed.

**Key Results:**

A binding pocket for VA at the β^+^/α^−^ interface encompassing amino acid β3N265 was predicted. Mutational analysis of suggested amino acid residues revealed a complete loss of VA's activity on β3M286W channels as well as significantly decreased efficacy and potency of VA on β3N265S and β3F289S receptors. In addition, reduced efficacy of VA‐induced *I*_GABA_ enhancement was also observed for α1M235W, β3R269A and β3M286A constructs.

**Conclusions and Implications:**

Our data suggest that amino acid residues β3N265, β3F289, β3M286, β3R269 in the β3 subunit, at or near the etomidate/propofol binding site(s), form part of a VA binding pocket. The identification of the binding pocket for VA is essential for elucidating its pharmacological effects and might also help to develop new selective GABA_A_ receptor ligands.

**Abbreviations**
TM: transmembrane
VA: valerenic acid

## Tables of Links

**Table:** 

**TARGETS**
GABAA receptors

**LIGANDS**		
[3H]‐azietomidate	Etomidate	Ivermectin
Diazepam	GABA	Propofol

These Tables list key protein targets and ligands in this article which are hyperlinked to corresponding entries in http://www.guidetopharmacology.org, the common portal for data from the IUPHAR/BPS Guide to PHARMACOLOGY (Pawson *et al*., 2014) and are permanently archived in the Concise Guide to PHARMACOLOGY 2013/14 (Alexander *et al*., 2013).

## Introduction

Valerenic acid (VA) – a sesquiterpenoid compound found in common valerian – allosterically modulates GABA_A_ receptors and induces anxiolysis and anticonvulsant effects (Khom *et al*., 2007; Benke *et al*., 2009; Hintersteiner *et al*., 2014).

GABA_A_ receptors are the major inhibitory neurotransmitter receptors in the mammalian brain (Olsen and Sieghart, 2008; Sigel and Steinmann, 2012) and regulate the sleep‐wake cycle, mood and emotions as well as seizure susceptibility (Möhler, 2006, 2012; Crow, 2013). GABA_A_ receptors are constituted by a pseudosymmetrical assembly of five identical or homologous subunits forming a chloride‐conducting ion pore (Tretter *et al*., 1997; Baumann *et al*., 2002; Baur *et al*., 2006; Sigel *et al*., 2006). Each subunit comprises a 200‐ to 250‐amino acids‐long extracellular N‐terminal domain, a loose bundle of four membrane‐spanning α‐helices (TM1–TM4), a large intracellular loop between the TM3 and TM4 domain (between 85 and 255 amino acid residues) and a short C‐terminal segment. Residues from the TM2 domain line the ion‐conducting pore (Olsen and Tobin, 1990; Olsen and Sieghart, 2008; Miller and Aricescu, 2014).

In the human genome, genes encoding for 19 different GABA_A_ receptor subunits belonging to eight families (α1‐6, β1‐3, γ1‐3, δ, ε, ρ1‐3, π and θ) have been identified (Simon *et al*., 2004). The subunit composition determines the pharmacological profile of the receptor (Olsen and Sieghart, 2008, 2009).

VA selectively interacts with a subset of GABA_A_ receptors comprising β2 or β3 subunits while displaying negligible effects on β1‐containing channels. At high concentrations, VA directly activates (≥30 μM) and inhibits (≥100 μM) GABA_A_ receptors (Khom *et al*., 2007).

A single asparagine residue (β2/3N265) in the pore‐lining TM2 was identified as a key determinant for VA's *I*_GABA_ enhancement *in vitro* (Khom *et al*., 2007) and its anxiolytic activity in mice (Benke *et al*., 2009). This residue (β2/3N265) is also essential for subunit‐selective modulation of GABA_A_ receptors by drugs such as etomidate (Belelli *et al*., 1997; Jurd *et al*., 2003; Stewart *et al*., 2014), loreclezole (Wafford *et al*., 1994; Wingrove *et al*., 1994; Groves *et al*., 2006) and mefenamic acid (Halliwell *et al*., 1999). While transmembrane (TM) binding pockets for etomidate (Li *et al*., 2006; Olsen and Li, 2011; Chiara *et al*., 2013), propofol (Chiara *et al*., 2013; Jayakar *et al*., 2014), barbiturates (Chiara *et al*., 2013) and neurosteroids (Hosie *et al*., 2006, 2007, 2009; Chen *et al*., 2012) have been identified, the localization of the binding site of VA on GABA_A_ receptors is still unknown.

VA was docked into a pocket at the β^+^/α^−^ interface encompassing amino acid residue β265 of α1β1/3γ2S GABA_A_ receptor homology models based on the glutamate‐gated chloride channel (3RIF; Hibbs and Gouaux, 2011), and a structure‐based three‐dimensional (3D) pharmacophore was designed. These studies suggested direct interactions between VA's carboxylate group and residues β3N265/β1S265 and β1/3R269 as well as multiple hydrophobic contacts to the lipophilic pocket surface. In order to test this hypothesis, selected amino acid residues of this pocket were mutated, and the enhancement of *I*_GABA_ by VA through mutant and wild‐type channels expressed in *Xenopus* oocytes was analysed.

## Methods

### Groups sizes

Numbers (*n*) for all experiments are provided and refer to independent single measurements. Data subjected to statistical analysis have *n* of at least 5 per group.

### Randomization

Oocytes were harvested from randomly selected frogs. To ensure reproducibility, wild‐type and mutant receptors were expressed and studied in batches of oocytes from at least two different frogs.

### Blinding

Experiments, when and where applicable, were performed and analysed by at least two different operators and the identity of the receptor subtype studied only revealed after the data set had been completed.

### Normalization

Stimulation of GABA‐induced chloride currents (*I*_GABA_) by VA was measured at a GABA concentration eliciting between 3 and 7% of the maximal current amplitude (EC_3–7_). The EC_3–7_ was determined at the beginning of the experiment for each oocyte by application of 1–3 mM GABA followed by submaximal GABA concentrations. Enhancement of the chloride current was defined as (I_(GABA + Comp)_/I_GABA_) − 1, where I_(GABA + Comp)_ is the current response in the presence of compound and I_GABA_ is the control GABA current. Concentration‐response curves were generated, and the data were fitted by non‐linear regression analysis using origin software (OriginLab Corporation, Northampton, MA, USA). Data were fitted to the Hill equation:

$$y\;=\;\min\;+\;\left(\max-\min\right)\;*\;x^{n}/\left(k^{n_{H}}\;+\;x^{n_{H}}\right)$$

*k* corresponds to the EC_50_ value; *x*‐values are logs of concentration, and *n*_H_ is the Hill coefficient. Each data point represents the mean ± SEM from ≥3 oocytes and two oocyte batches.

### Validity of animal species or model selection

*Xenopus* oocytes are widely accepted as a model system for the expression of ion channels and studies on ion channel pharmacology.

### Ethical statement

All experiments involving animals were approved by the Austrian Animal Experimentation Ethics Board in compliance with the European convention for the protection of vertebrate animals used for experimental and other scientific purposes ETS no. 123, which is in line with the EU Directive 2010/63/EU (GZ 66.006/0019‐C/GT/2007). All studies involving animals are in accordance with the ARRIVE guidelines for reporting experiments involving animals (Kilkenny *et al*., 2010; McGrath *et al*., 2010).

### Animals

12 female African claw frogs (*Xenopus laevis*; approximate age 1 year; weight between 200 and 250 g) purchased from NASCO (Fort Atkinson, WI, USA) were used in the present study.

### Experimental procedures

Frogs were anaesthetized by exposing them to a 0.2% solution of MS‐222 (methane sulfonate salt of 3‐aminobenzoic acid ethyl ester) for 15 min before surgically removing parts of the ovaries (0.5 to 1 cm abdominal incision). After surgery, frogs were allowed to recover for at least 6 months. Animals were not killed for experimental procedures.

### Housing and husbandry

Frogs were kept in groups (max. eight per tank) in a temperature‐controlled and humidity‐controlled animal facility (20 ± 2°C; 50 ± 10%) in continuous‐flow water tanks (water temperature fixed at 20 ± 1°C; tank shape > 30 × 50 × 60 cm).

### Interpretation

Every effort was taken to minimize the number of animals used in this study.

#### Homology modelling and docking

GABA_A_ receptor α1β3γ2S and α1β1γ2S homology models were generated on the basis of an ivermectin‐bound structure of the glutamate‐gated chloride channel structure 3RIF (Hibbs and Gouaux, 2011) and on the basis of the recently released GABA_A_ receptor β3‐homopentameric crystal structure 4COF (Miller and Aricescu, 2014) using the modeller software (Sali and Blundell, 1993).

Docking studies were performed with AutoDock4 (Morris *et al*., 2009). Homology models of GABA_A_ receptors and VA were opened in AutoDockTools. AutoDock4 atom types were assigned, and Gasteiger charges of all structures were computed and then saved as. pdbqt files.

A grid box (grid points of 40 × 40 × 40 with a spacing of 0.375 Å) was centred on the potential pocket defined by the centrally located β3N265 and β1S265.

Flexible docking studies were performed on α1β3γ2S and α1β1γ2S models where α1I227, α1M235, β3N265 (β1S265), β1/3M286, β1/3F289 and VA were kept flexible during docking runs. As a result, 1000 runs were generated, to ensure convergence of the sampling.

Refinement of the highest ranked docking poses and analysis of VA interactions with the protein environment of the binding sites was performed by the pharmacophore modelling software ligandscout 4.04 (Wolber and Langer, 2005). Within ligandscout, binding sites on the α1β1γ2S and α1β3γ2S receptor were defined using residues α1I227, α1M235, β3N265/β1S265, β1/3M286 and β1/3F289 as anchor points. The selected docking poses of VA were then inserted into the respective binding sites and structure optimized with the MMFF94 force field (stopping criterion: root square square (RMS) gradient ≤0.1). During the energy optimization run, the ligand VA and amino acid side chains were allowed to move, and the atoms of the protein backbone were kept fixed. Analysis of the interactions of VA with the binding pockets' amino acids was carried out by generation of a structure‐based 3D pharmacophore using the previously optimized VA pose and side chains as input.

#### Expression of wild‐type and mutant GABA_A_ receptors in *Xenopus laevis* oocytes

Follicle membranes covering oocytes were enzymatically digested with 2 mg·mL^−1^ collagenase (type 1A). Mutations β3T262A, β3T262S, β3N265S, β3T266A, β3R269A, β3M286A, β3M286W and β3F289S in the β3‐subunit and α1I227A, α1L231A, α1M235A, α1M235W and α1L268A in the α1 subunit were introduced by site‐directed mutagenesis using the QuikChange mutagenesis kit (Agilent Technologies, Vienna, Austria). The coding regions of plasmids were sequenced before experimental use. After cDNA linearization, capped cRNA transcripts were produced using the mMESSAGE mMACHINE® T7 transcription kit (Life Technologies). Capped transcripts were polyadenylated using yeast poly(A)polymerase, diluted in nuclease‐free water and stored before injection at −80°C.

One day after isolation, the oocytes were injected with about 10–50 nL of nuclease‐free water containing the different rat cRNAs (100–2000 ng·μL^−1^ per subunit). For expression of wild‐type α1β3γ2S and mutant receptors, cRNAs were mixed in a ratio of 1:1:10 (Boileau *et al*., 2002). Electrophysiological experiments were performed using the two‐microelectrode voltage clamp technique at a holding potential of −70 mV making use of a TURBO TEC 01C amplifier (NPI Electronic, Tamm, Germany) and an Axon Digidata 1322A interface (Molecular Devices, Sunnyvale, CA, USA). Data acquisition was carried out using pclamp v.9.2 (Molecular Devices, Sunnyvale, CA). The bath solution contained 90 mM NaCl, 1 mM KCl, 1 mM MgCl_2_, 1 mM CaCl_2_ and 5 mM HEPES (adjusted to pH 7.4 using 1 M NaOH). Microelectrodes were filled with 2 M KCl and had resistances between 1 and 3 MΩ (Khom *et al*., 2006).

#### Perfusion system

GABA and VA were applied by means of a fast perfusion system (for details, see Baburin *et al*., 2006). Drug or control solutions were applied by means of a TECAN Miniprep 60 permitting automation of the experiments. To elicit *I*_GABA_, the chamber was perfused with 120 μL of GABA‐containing solution at a volume rate between 300 and 1000 μL·s^−1^. To account for possible slow recovery from increasing levels of desensitization in the presence of high GABA or compound concentrations, the duration of washout periods was extended stepwise, that is, 1.5 min (control GABA EC_3–7_), 3 min (co‐application of GABA EC_3–7_ in the presence of ≤1 μM VA), 5–10 min (co‐application of GABA EC_3–7_ in the presence of 3–30 μM VA) and ≥15 min (co‐application of GABA EC_3–7_ and 100–500 μM VA). Potential run‐down or run‐up effects were ruled out by application of GABA control at the end of each experiment. Oocytes with maximal current amplitudes >5 μA were discarded to exclude voltage‐clamp errors (Khom *et al*., 2006).

### Statistical comparison

Statistically significant differences were calculated using one‐way ANOVA followed by a *post hoc* mean comparison (Dunnett; GraphPad, La Jolla, CA, USA) using independent measurements. Only *P*‐values <0.05 were accepted as statistically significant.

#### Chemicals

All chemicals used in this study were obtained from Sigma Aldrich (Vienna, Austria) except VA, which was purchased from HWI Pharma Solutions (Rülzheim, Germany) and where stated otherwise; 100 mM stock solutions of VA were prepared in 100% dimethyl sulfoxide (DMSO). VA was used up to a concentration of 500 μM. Equal amounts of DMSO were present in control and compound‐containing solutions. The maximum DMSO concentration present in the bath (0.5%) did not affect *I*_GABA_.

## Results

### Computational studies

In order to determine whether the region around residue β3N265 would be suited to contain VA's binding pocket, VA was initially docked into two different α1β3γ2S GABA_A_ receptor homology models based on the glutamate‐gated chloride channel 3RIF (Hibbs and Gouaux, 2011) and the recently released β3‐homopentameric GABA_A_ receptor crystal structure 4COF (Miller and Aricescu, 2014). The putative pocket was defined by a cut‐off distance of 10 Å around residue β3N265. According to Ligplot analysis (Wallace *et al*., 1995) of an initial docking experiment, poses with more protein–ligand interactions were obtained from the 3RIF‐based model. It may seem surprising that models based on the more remote homologue (glutamate‐gated chloride channel) perform better than those based on the recently crystallized β3‐GABA_A_ receptor homopentamer. However, closer analysis revealed that the 3RIF template, which has an ivermectin molecule bound in a pocket homologous to the herewith proposed VA pocket, is in a ligand‐bound conformation, while 4COF has no ligand bound to this particular pocket. Thus, we only considered the 3RIF‐based models for further docking studies.

**Figure 1:**
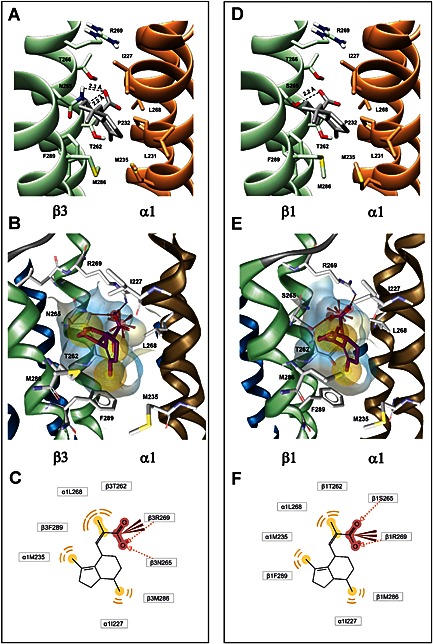
Putative binding pocket(s) of VA located at the β^+^/α^−^ interface on GABA_A_ receptors and two‐dimensional representations of VA are shown. The α1 subunit is coloured in brown, and the respective β subunits (β3 in (A, B) and β1 in (D, E)) are shown in green. VA and interacting amino acid side chains are shown in stick rendering, colour coded as to atom type: red, oxygen; dark blue, nitrogen; yellow, sulfur. Top row: VA poses derived from docking: TM residues β1/3T262, β3N265/β1S265, β1/3T266, β1/3R269, β1/3M286 and β1/3F289 and α1I227, α1L231, α1P232, α1M235 and α1L268 are pocket‐defining or very close to the pocket. Energetically, most favourable orientations of VA obtained from docking into (A) α1β3γ2S and (D) α1β1γ2S GABA_A_ receptor homology models are illustrated. Dashed lines indicate distances between VA's carboxylate group and putative H‐bond interaction partners on β3N265 (A) and β1S265 (D) respectively. Middle row: Refined poses and resulting putative pharmacophore: (B, E) Optimized docking poses of VA (marked in red/ purple) in the β3^+^/α1^−^ and β1^+^/α1^−^ binding pockets are shown. All surrounding amino acid side chains were kept flexible. Strong changes in rotamers compared with the docking results shown in the top row can be observed for β1/3R269 and β1/3F289. The structure‐based pharmacophore features three lipophilic contacts (yellow spheres), two putative H‐bond acceptor interactions (red arrows) and one putative ionic interaction (red star). All amino acids that interact with the ligand are highlighted in a stick display style. Bottom row: Two‐dimensional rendering of the structure‐based pharmacophores: (C, F) Schematic two‐dimensional representations of the structure‐based pharmacophores of VA in the proposed binding pockets are shown. The carboxyl group forms an H bond with the –NH_2_ group of β3N265, or the –OH group of β1S265S. Additionally, the guanidinium group of β1/3R269 could form ionic or H‐bonding interactions with the carboxylate group. Hydrophobic interactions occur between VA's three methyl groups and the side chains of α1I227, α1M235, α1L268, β1/3T262, β1/3M286 and β1/3F289, which form the lipophilic part of the binding pocket surface.

To ensure convergence of the sampling, flexible docking studies with 1000 genetic algorithm runs were then performed using the 3RIF‐based α1β1γ2S and α1β3γ2S GABA_A_ receptor homology models with flexible side chains (α1I227, α1M235, β3N265 (β1S265), β1/3M286, β1/3 F289) in AutoDock4 (Morris *et al*., 2009). The best docking poses with the lowest estimated binding free energy scores in the top clusters for both α1β1γ2S (ΔG: −14.68 kcal·mol^−1^) and α1β3γ2S (ΔG: −14.99 kcal·mol^−1^) models were strictly selected with the root mean squared deviations criteria of 1 Å. Distance measurements suggest that H bonds are formed between VA's carboxylate and β3N265 (2.2–2.3 Å) and β1S265 (2.2 Å) respectively (Figure 1A and 1D). In addition, hydrophobic contacts presumably include side chains from amino acid residues α1I227, α1L231, α1P232, α1M235, β1/3M286 and β1/3F289 in both α1β1γ2S and α1β3γ2S models forming the hydrophobic pocket surface (Figure 1A and 1D).

To provide additional evidence for the validity of the assumptions that H bonding and hydrophobic contacts play a crucial role in VA binding, the previously selected best docking poses were further refined and analysed with ligandscout 4.04 (Wolber and Langer, 2005) using the following workflow: The PDB files for the α1β1γ2S and α1β3γ2S receptor were opened in ligandscout, and an active site was defined that included the residues α1I227, α1M235, β3N265/β1S265, β1/3M286 and β1/3F289. The selected docking poses of VA were then inserted into the corresponding binding sites and optimized with the MMFF94 force field. During optimization, both the ligand and the side chains of the amino acids were kept flexible. Considerable changes in side chain rotamers result during optimization (Figure 1). For analysis of the actual interactions of VA with the binding pockets' amino acids, a structure‐based pharmacophore was created. The models and refined poses of VA for both subunits obtained are displayed in Figure 1B and 1E, while Figure 1C and 1F provide schematic two‐dimensional representations of the interaction patterns.

In both the unrefined and refined binding pockets, the residues β1/3T262, β1/3M286, β1/3F289, α1M235, α1I227 and α1L268 are involved in the hydrophobic contacts of VA with the receptor surface. An H bond is formed with the –OH group of S265 in β1‐containing receptors and with the –NH_2_ of the amide group of N265 in β3‐containing receptors. In addition, the structure‐based 3D pharmacophore resulting from the optimized poses suggested an additional potential binding determinant, namely β1/3R269, potentially forming ionic or H‐bonding interactions with VA's carboxylate group. While the details of the binding mode might differ if a slightly different workflow in the computational analysis is chosen (such as different software packages, or different input parameters), the main aim here was to generate hypotheses that can be tested experimentally. Of interest thus is the final list of amino acids derived from both the docked poses and the refined poses that potentially interact with the ligand. The raw poses feature interactions with β1/3T262, β3N265/β1S265, β1/3T266, β1/3M286 and β1/3F289 and α1I227, α1L231, α1P232, α1M235 and α1L268. The refined poses and the resulting pharmacophores show no interactions of VA with β1/3T266, α1L231 or α1P232, and as new feature, they do display interactions with β1/3R269. Consequently, we selected the sum of putative interacting residues from both models for an experimental investigation.

### Expression and functional characterization of mutant GABA_A_ receptors

**Figure 2:**
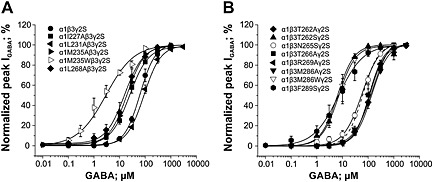
GABA concentration‐response curves for wild‐type α1β3γ2S and the mutant GABA_A_ channels indicated are compared. Panel (A) illustrates the effect of mutations of the α1 subunit (co‐expressed with β3 and γ2S subunits) on GABA sensitivity compared with wild‐type α1β3γ2S channels, while in panel (B), the impact of the β3 mutations on the GABA‐concentration response relation is shown (wild type illustrated as dashed line). Responses at indicated concentrations in each cell were normalized to the maximum GABA‐evoked peak current. Each data point represents the mean ± SEM of ≥5 oocytes from at least two batches.

In order to investigate the predicted binding pocket, point mutations were individually introduced (β3T262, β3N265, β3T266, β3R269, β3M286, β3F289, α1I227, α1L231, α1M235 and α1L268), and mutant constructs were co‐expressed with either wild‐type α1 or wild‐type β3 and γ2S subunits in *Xenopus laevis* oocytes. As illustrated in Figure 2, all mutants formed functional GABA‐gated chloride channels. Comparison of GABA concentration‐response curves for wild‐type α1β3γ2S (EC_50_ = 61.9 ± 2.1 μM; *n* = 7) and mutant channels revealed that only mutation β3N265S did not affect GABA sensitivity (EC_50_ = 57.2 ± 4.5 μM; *n* = 6), while the other mutations shifted the GABA‐concentration response curves either to the left or to the right. Increased GABA sensitivity was observed for mutant channels containing α1I227A, α1M235A, α1M235W, α1L268A, β3T262S, β3M286W and β3F289S subunits, while channels containing α1L231A, β3T262A, β3T266A, β3R269A and β3M286A subunits were characterized by rightward shifts of the GABA concentration‐response curve (see Figure 2A and B for GABA concentration‐response curves. EC_50_ values, Hill coefficients (*n*_H_) and number of experiments for the respective subunit composition are given in Table 1).

**Table 1:** Pharmacological properties of wild‐type α1β3γ2S and mutant GABA_A_ receptors

Subunit composition	EC_50_ (μM)	*n*_H_	*n*
α1β3γ2S	61.9 ± 2.1	1.44 ± 0.03	7
α1I227Aβ3γ2S	24.3 ± 1.4 ^***^	1.18 ± 0.04	5
α1L231Aβ3γ2S	89.1 ± 6.6 ^**^	1.32 ± 0.07	5
α1M235Aβ3γ2S	19.4 ± 2.2 ^***^	1.25 ± 0.13	6
α1M235Wβ3γ2S	2.9 ± 0.4 ^***^	0.79 ± 0.06	6
α1L268Aβ3γ2S	15.7 ± 1.9 ^***^	1.19 ± 0.1	5
α1β3T262Aγ2S	116.7 ± 9.1 ^***^	1.41 ± 0.06	5
α1β3T262Sγ2S	6.4 ± 0.1 ^***^	1.33 ± 0.14	6
α1β3N265Sγ2S	57.2 ± 4.5	1.23 ± 0.09	6
α1β3T266Aγ2S	143.7 ± 14.0 ^***^	1.21 ± 0.08	6
α1β3R269Aγ2S	108.4 ± 5.9 ^***^	1.51 ± 0.05	5
α1β3M286Aγ2S	138.4 ± 9.2 ^***^	1.33 ± 0.08	5
α1β3M286Wγ2S	7.1 ± 0.3 ^***^	1.33 ± 0.14	7
α1β3F289Sγ2S	7.0 ± 1.2 ^***^	0.90 ± 0.08	7

EC_50_ concentrations (μM) and Hill coefficients (*n*_H_) are given for each receptor as mean ± SEM for *n* number of cells tested. Statistical significance of difference from wild‐type was calculated using a one‐way ANOVA followed by a Dunnett's mean comparison test. ^**^ *P* < 0.01; ^***^ *P* < 0.001.

### Effects of point mutations in β3TM2, β3TM3, α1TM1 and α1TM2 domains on *I*_GABA_ enhancement by VA and diazepam

**Figure 3:**
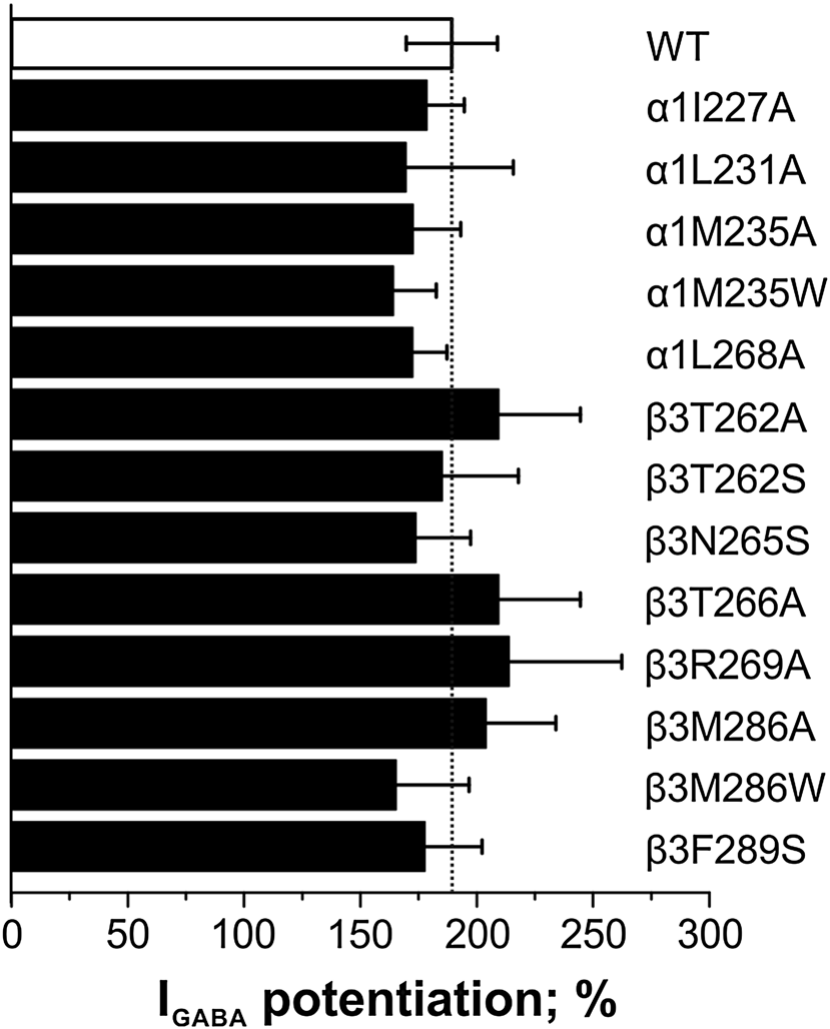
Potentiation of submaximal GABA responses (EC_3–7_) by 1 μM diazepam of mutant receptors (black bars) is compared with wild‐type α1β3γ2S receptors (white bar). Bars represent means ± SEM (*n* = 3 for α1L231Aβ3γ2S, α1M235Aβ3γ2S α1L268Aβ3γ2S, α1β3T262Aγ2S, α1β3R269Aγ2S and α1M286Aβ3γ2S; *n* = 4 for α1I227Aβ3γ2S; *n* = 5 for α1M235Wβ3γ2S and α1β3T266Aγ2S; *n* = 6 for α1β3γ2S, α1β3T262Sγ2S and α1β3F289Sγ2S; *n* = 7 for α1β3N265Sγ2S and α1β3286Wγ2S; cells were taken from at least two different oocyte batches).

Increase in GABA sensitivity (evident from a left shift of GABA‐concentration response curves of α1I227A, α1M235A, α1M235W, α1L268A, β3T262S, β3M286W and β3F289S mutants) may reflect a destabilization of the closed‐channel state relative to the open state (Figure 2, Table 1). This can reduce the ability of drugs to potentiate *I*_GABA_ (Bianchi and Macdonald, 2003; Stewart *et al*., 2008). However, similar *I*_GABA_ potentiation by diazepam (1 μM; Figure 3) indicates that all mutant receptors retained their responsiveness to this allosteric GABA_A_ receptor modulator irrespective of the changes in the GABA sensitivity of the mutants.

**Figure 4:**
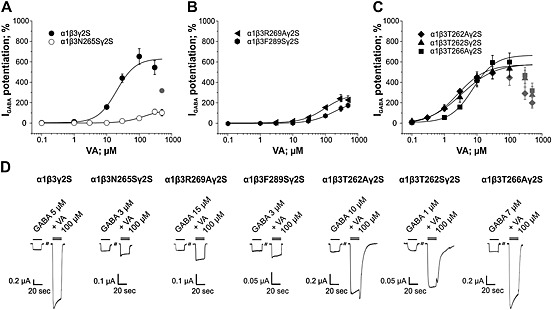
Effects of mutating residues β3N265, β3R269, β3F289, β3T262, and β3T266 on efficacy and potency of *I*_GABA_ enhancement by VA are shown. Concentration‐response curves for VA‐induced *I*_GABA_ enhancement on (A) α1β3γ2S, α1β3N265Sγ2S, (B) α1β3R269Aγ2S, α1β3F289Sγ2S, (C) α1β3T262Aγ2S, α1β3T262Sγ2S and α1β3T266Aγ2S receptors are illustrated. Responses in each cell were normalized to a submaximal GABA EC_3–7_ concentration determined at the beginning of each experiment. Data points represent the mean ± SEM of ≥5 oocytes from at least two batches. Error bars smaller than the symbol are not shown. Grey symbols are excluded from the fit. (D) Representative current traces in the presence of 20 s application of a GABA EC_3–7_ concentration (single bar) or co‐application of GABA EC_3–7_ and 100 μM VA recorded from *Xenopus laevis* oocytes voltage‐clamped at −70 mV expressing the indicated receptor subtype.

As illustrated in Figure 4A, VA potently and efficaciously enhanced *I*_GABA_ (GABA EC_3–7_) through α1β3γ2S receptors (EC_50_ = 20.2 ± 5.2 μM; E_max_ = 632 ± 88%; *n* = 9).

Mutation of amino acid residue β3N265 to serine (corresponding residue in β1 subunits) significantly reduced efficacy and potency of VA at enhancing the *I*_GABA_. Efficacy of I_GABA_ enhancement through α1β3N265Sγ2S was approximately fivefold reduced accompanied by a sevenfold reduction of potency (E_max_ = 134 ± 32%; EC_50_ = 142.9 ± 67.5 μM; *n* = 7; *P* < 0.001; Figure 4A and Table 2). Similarly, efficacy and potency of I_GABA_ modulation by VA through α1β3F289Sγ2S was significantly reduced compared with wild type (E_max_ = 222 ± 12%; EC_50_ = 180.6 ± 21.6 μM; *n* = 8; *P* < 0.001; Figure 4B, Table 2). A comparable loss of efficacy of *I*_GABA_ enhancement by VA was observed for α1β3R269Aγ2S receptors (E_max_ = 259 ± 22%; *n* = 7; *P*< 0.001). In addition, a trend towards decreased VA potency on this mutant was observed; however, this effect did not reach statistical significance (EC_50_ = 84.1 ± 14.7 μM; *P* > 0.05; Figure 4B).

**Table 2:** Parameters of *I*_GABA_ enhancement of wild‐type α1β3γ2S and mutant GABA_A_ receptors by VA

Subunit composition	E_max_ (%)	EC_50_ (μM)	*n*_H_	*n*
α1β3γ2S	632 ± 88	20.2 ± 5.2	1.50 ± 0.29	9
α1I227Aβ3γ2S	776 ± 60	40.8 ± 8.4	1.13 ± 0.08	6
α1L231Aβ3γ2S	703 ± 82	18.8 ± 4.4	1.37 ± 0.18	5
α1M235Aβ3γ2S	546 ± 28	21.1 ± 2.8	1.19 ± 0.08	6
α1M235Wβ3γ2S	193 ± 16 ^***^	24.3 ± 7.7	1.37 ± 0.34	6
α1L268Aβ3γ2S	582 ± 80	19.9 ± 5.6	1.06 ± 0.13	5
α1β3T262Aγ2S	573 ± 96	2.5 ± 0.8	1.00 ± 0.16	5
α1β3T262Sγ2S	578 ± 42	3.4 ± 0.7	0.85 ± 0.15	9
α1β3N265Sγ2S	134 ± 32 ^***^	142.9 ± 67.5 ^***^	1.33 ± 0.39	7
α1β3T266Aγ2S	666 ± 55	7.5 ± 1.5	1.26 ± 0.1	12
α1β3R269Aγ2S	259 ± 22 ^***^	84.1 ± 14.7	1.63 ± 0.19	7
α1β3M286Aγ2S	283 ± 52 ^***^	60.1 ± 18.5	1.47 ± 0.21	6
α1β3M286Wγ2S	67 ± 35 ^***^	n.d.	n.d.	10
α1β3F289Sγ2S	222 ± 12 ^***^	180.6 ± 21.6 ^***^	1.20 ± 0.07	8

Maximal efficacies (E_max_, %), EC_50_ concentrations (μM) and Hill coefficients (*n*_H_) are given for each receptor as mean ± SEM for *n* number of cells tested. Statistical significance of difference from wild‐type was calculated using a one‐way ANOVA followed by Dunnett's mean comparison test. n.d., non‐determined. ^***^ *P* < 0.001.

In contrast, no effect on efficacy of *I*_GABA_ enhancement by VA was observed upon mutating two threonine residues adjacent to β3N265 (β3T262S, β3T262A and β3T266A). However, even though not statistically significant (*P* > 0.05), a trend towards increased potency of VA on these mutant receptors was observed (Figure 4C; see also Table 2).

**Figure 5:**
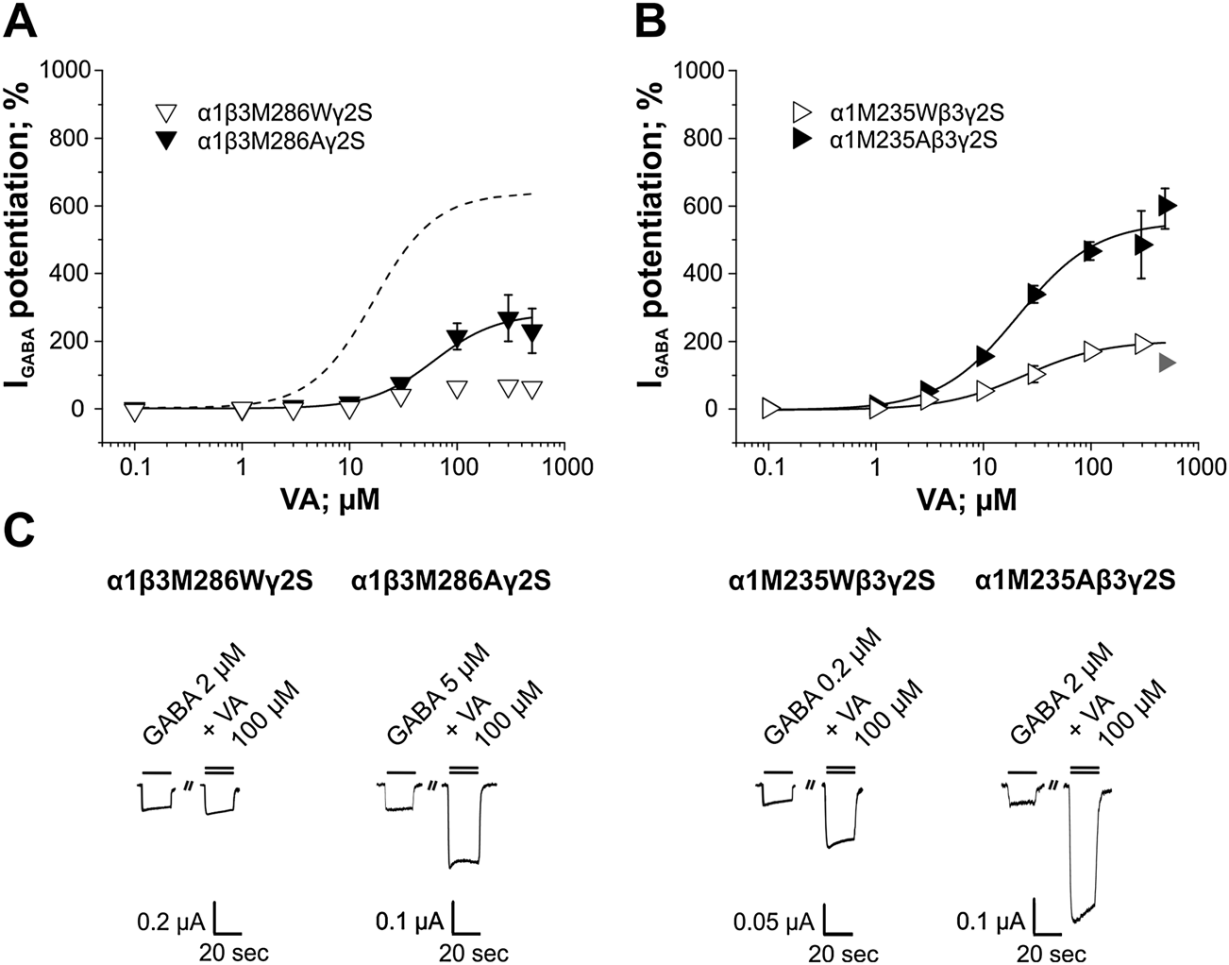
Effects of mutating residues β3M286 and α1M235 on efficacy and potency of *I*_GABA_ enhancement by VA are illustrated. Concentration‐response curves for VA‐induced *I*_GABA_ enhancement on (A) α1β3M286Wγ2S and α1β3M286Aγ2S and (B) α1M235Wβ3γ2S and α1M235Aβ3γ2S receptors are shown. Dashed line in (A) represents *I*_GABA_ enhancement by VA on wild‐type channels. Responses in each cell were normalized to a submaximal GABA EC_3–7_ concentration determined at the beginning of each experiment. Data points represent the mean ± SEM of ≥6 oocytes from at least two batches. Error bars smaller than the symbol are not shown. Grey symbols are excluded from the fit. (C) Representative current traces in the presence of 20 s application of a GABA EC_3–7_ concentration (single bar) or co‐application of GABA EC_3–7_ and 100 μM VA recorded from *Xenopus laevis* oocytes voltage‐clamped at −70 mV expressing the indicated receptor subtype.

Two amino acid residues previously photolabelled by the etomidate analogue [^3^H]‐azietomidate (β3M286 and α1M235; Li *et al*., 2006; Stewart *et al*., 2008) seem to contribute to VA's interaction with GABA_A_ receptors (Figure 1). Like etomidate, VA did not display any significant modulatory effects on *I*_GABA_ through α1β3M286Wγ2S receptors; efficacy of *I*_GABA_ enhancement through α1M235Wβ3γ2S (E_max_ = 193 ± 16%, *n* = 6, *P* < 0.001; Figure 5A) receptors was also significantly reduced compared with wild‐type α1β3γ2S channels. In addition, efficacy of *I*_GABA_ enhancement through α1β3M286Aγ2S by VA was also significantly reduced efficacy compared to wild‐type α1β3γ2S receptors (E_max_ = 283 ± 52%; *P*< 0.001; EC_50_ = 60.1 ± 18.5 μM; *n* = 6; *P* > 0.05; Figure 5B). Most notably, enhancement of *I*_GABA_ by VA through α1M235Aβ3γ2S receptors did not differ from wild‐type in terms of efficacy and potency. Representative currents illustrating the effect of mutations β3M286W/A and α1M235W/A on *I*_GABA_ enhancement are shown in Figure 5C.

**Figure 6:**
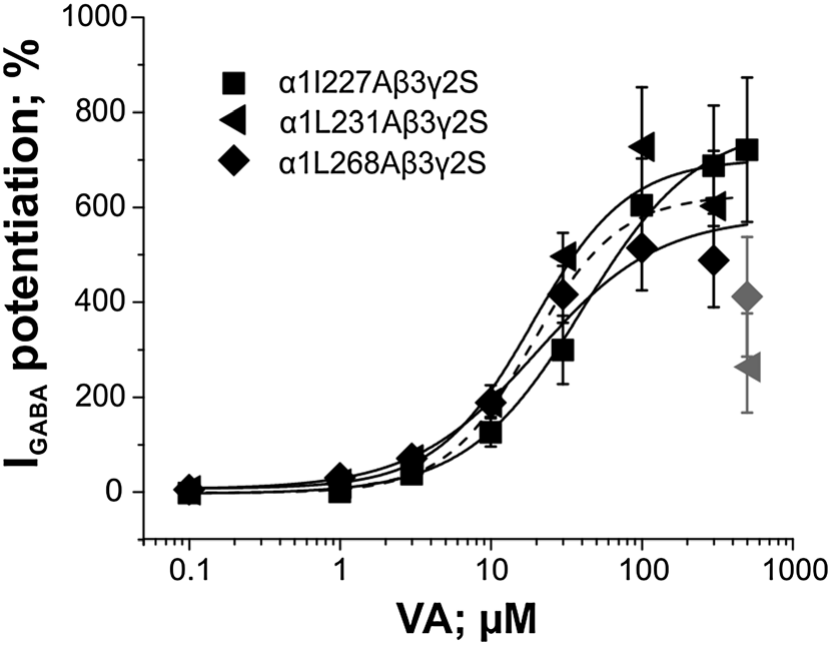
Concentration‐response curves for VA‐induced *I*_GABA_ enhancement on α1I227Aβ3γ2S, α1L231Aβ3γ2S and α1L268β3γ2S receptors. Dashed line represents *I*_GABA_ enhancement by VA on wild‐type channels. Responses in each cell were normalized to a submaximal GABA EC_3–7_ concentration determined at the beginning of each experiment. Data points represent the mean ± SEM of ≥5 oocytes from at least two batches. Error bars smaller than the symbol are not shown. Grey symbols are excluded from the fit.

As illustrated in Figure 6, no effect on *I*_GABA_ enhancement was observed upon mutating amino acid residues α1I227, α1L231 and α1L268. *I*_GABA_ enhancement by VA through α1I227Aβ3γ2S, α1L231Aβ3γ2S and α1L268β3γ2S receptors did not differ significantly from wild‐type α1β3γ2S either in terms of efficacy or potency (see also Table 2).

## Discussion

VA selectively modulates GABA_A_ receptors containing β2/3 subunits, while only a small enhancement of β1‐containing receptors is observed (Khom *et al*., 2007). Similar to other β2/3‐selective GABA_A_ ligands including etomidate (Jurd *et al*., 2003; Stewart *et al*., 2014), loreclezole (Wafford *et al*., 1994; Wingrove *et al*., 1994; Groves *et al*., 2006) or mefenamic acid (Halliwell *et al*., 1999), VA's subunit selectivity is determined by the presence of an asparagine residue in the TM2 domain (β2/3N265). Mutation of β2/3N265 to either serine (corresponding amino acid residue in the β1 subunit; Khom *et al*., 2007) or methionine (Benke *et al*., 2009) results in drastically reduced sensitivity for VA‐induced *I*_GABA_ enhancement.

In order to localize VA's binding pocket, the molecule was docked into α1β1γ2S and α1β3γ2S GABA_A_ receptor homology models based on the glutamate‐gated chloride channel (3RIF; Hibbs and Gouaux, 2011), suggesting a common binding pocket for VA located at the β^+^/α^−^ subunit interface encompassing residue β3N265/β1S265.

In order to validate the proposed pocket for VA on GABA_A_ receptors on the β^+^/α^−^ interface, selected amino acid residues from both α1 and β3 subunits were mutated, expressed in *Xenopus* oocytes with either wild‐type α1 or β3 subunits and a γ2S‐subunit, and VA‐induced enhancement of *I*_GABA_ through mutant and wild‐type receptors was compared. Our docking studies predicted that VA's carboxylate forms an H bond to β3N265/β1S265, putative ionic or H bond interactions with β1/3R269 and multiple hydrophobic interactions with the pocket's lipophilic surface. Indeed, mutating amino acid residue β3N265 to the corresponding serine residue in β1 (β3N265S) nearly abolished VA‐induced I_GABA_ enhancement (approximately fivefold‐reduced efficacy and sevenfold‐reduced potency). Furthermore, mutating the arginine β3R269 to alanine reduced the efficacy of *I*_GABA_ enhancement by approximately 50%. This mutation, however, did not significantly affect VA potency (Figure 4B). Whether this mutation interrupts an ionic interaction of VA with βR269 (Figure 1) or induces other changes in the putative binding pocket warrants further studies.

Apart from these interactions, several hydrophobic interactions of amino acid residues located in both α1 and β3 subunits with VA were suggested to contribute to efficacious and potent *I*_GABA_ enhancement.

Mutating amino acid residues β3M286 and β3F289 significantly reduced the efficacy of *I*_GABA_ enhancement by VA (see Figure 4B for VA action on α1β3F289Sγ2S channels and 5A on α1β3M286Aγ2S). The reduction in efficacy for VA in the case of α1β3F289Sγ2S channels was also accompanied by a significant rightward shift of VA's potency (ninefold).

In contrast, removal of other potential hydrophobic interactions by introducing alanine residues (α1I227, α1L231, α1M235, α1L268, β3T262, β3T266) did not significantly alter the *I*_GABA_ enhancement (Figures 4C, 5B and 6), suggesting that loss of single hydrophobic interactions might be well tolerated or even compensated for by other amino acid residues from the lipophilic surface of the binding pocket. However, introducing a bulky residue in position β3M286 resulted in a complete loss of VA's action (Figure 5A). We speculate that such a substitution might occlude the entrance and/or reduce the volume of VA's binding pocket. Similar results have been previously reported for etomidate (Stewart *et al*., 2008).

Reduced drug efficacy observed on mutant channels may also result from altered channel gating. Leftward shifts of the GABA‐concentration response curve were observed for seven of the mutants studied (Figure 2 and Table 1), indicating that these mutations might destabilize the closed state of the channel relative to the open state (Bianchi and Macdonald, 2003; Stewart *et al*., 2008), which could compromise *I*_GABA_ modulation. However, similar *I*_GABA_ potentiation by diazepam (Figure 3) would argue for retained responsiveness to modulators. This is also nicely demonstrated by mutations in positions β3T262 causing either a left‐ or rightward shift in GABA sensitivity without affecting *I*_GABA_ modulation by VA.

Possible effects of VA at other homologous pockets, specifically at the γ^+^/β^−^, α^+^/γ^−^ and α^+^/β^−^ interface, have not been investigated explicitly. Inspection of homologous TM pockets in the GABA_A_ receptor model other than the β^+^/α^−^ subunit interface revealed that the potential strong binding determinant βN265 at the β^+^/α^−^ interface has serine residues in the homologous position at all other interfaces (γ2S280 at the γ^+^/β^−^, α1S269 at α^+^/γ^−^ and α^+^/β^−^ interface, respectively). Furthermore, the homologous position of β3M286 (shown to be essential for efficacious *I*_GABA_ enhancement by VA; see also Figure 5A for the effect of mutating this residue to alanine in the β^+^/α^−^ interface) at the β^+^/α^−^ interface is an alanine in α1 (α1A290) at the α^+^/β^−^ and at α^+^/γ^−^ interfaces, and a serine residue at the γ^+^/β^−^ interface (γ2S301). Considering the essential role of residue N265 in the β^+^/α^−^ subunit interface for efficacious and potent *I*_GABA_ enhancement by VA and the observed loss of efficacy/potency when β2/3N265 is mutated to serine, which naturally occurs in β1‐containing receptors (Khom *et al*., 2007; Benke *et al*., 2009) and the loss of efficacy when β3M286 is mutated to alanine (Figure 5B), we consider an interaction of VA with homologous binding pockets at other subunit interfaces unlikely.

## Conclusion

Although a participation in transduction of gating effects cannot be excluded, our computational and experimental data suggest that amino acid residues β3N265, β3R269, β3M286 and β3F289, at or near the etomidate/propofol binding site(s), form part of a VA binding pocket. Further mutational and computational studies will focus on the identification of additional potential binding determinants within the proposed pocket and the mechanism by which VA modulates the GABA_A_ receptor; this might also help in the development of new selective GABA_A_ receptor ligands.

## Author contributions

D L performed research, designed the research study, analysed data and wrote the paper. G P performed research and analysed data. M W performed research and analysed data. M S performed research and analysed data. S K performed research and analysed data. M E performed research, analysed data and contributed to writing the paper. A H contributed research tools. T L performed research and analysed data. T S performed research, analysed data and contributed to writing the paper. T L performed research, analysed data and contributed to writing the paper. S K designed the research study and wrote the paper. S H designed the research study and wrote the paper.

## Conflict of interest

S K and S H are inventors of patents EP 2389350 A1 20111130 and US 8809395 B2.
